# Case Report: Stüve–Wiedemann syndrome—a rare cause of persistent pulmonary hypertension of the newborn

**DOI:** 10.3389/fped.2023.1329404

**Published:** 2024-01-04

**Authors:** Jessica Jin, Paula Rothämel, Johanna Büchel, Birgit Kammer, Theresa Brunet, Joseph Pattathu, Andreas W. Flemmer, Claudia Nussbaum, Sebastian Schroepf

**Affiliations:** ^1^ Division of Neonatology, Department of Pediatrics, Dr. von Hauner Children’s Hospital, University Hospital, Ludwig-Maximilians-Universität, Munich, Germany; ^2^ Clinic and Outpatient Clinic for Obstetrics and Gynecology, University Hospital, Ludwig-Maximilians-Universität, Munich, Germany; ^3^ Department of Pediatric Radiology, Dr. von Hauner Children’s Hospital, University Hospital, Ludwig-Maximilians-Universität, Munich, Germany; ^4^ Institute of Human Genetics, Klinikum Rechts der Isar, University Hospital, Technical University of Munich, Munich, Germany; ^5^ Department of Pediatric Cardiology and Pediatric Intensive Care, University Hospital, Ludwig-Maximilians-Universität, Munich, Germany

**Keywords:** case report, Stüve–Wiedemann syndrome, PPHN, limb abnormalities, whole exome sequencing

## Abstract

**Introduction:**

Persistent pulmonary hypertension of the newborn (PPHN) is a life-threatening condition characterized by hypoxemia due to elevated pulmonary vascular resistance. PPHN commonly arises secondary to various underlying conditions, including infection, meconium aspiration, and respiratory distress syndrome. Management includes pulmonary vasodilators, mechanical ventilation, oxygen supplementation, vasopressors, and volume replacement. Stüve–Wiedemann syndrome (SWS), a rare genetic disorder characterized by bone dysplasia, respiratory distress, hyperthermia, and swallowing difficulties, may present with pulmonary hypertension, indicating a poor prognosis.

**Case description:**

A term female neonate presented with secondary respiratory failure and severe PPHN of unknown etiology on the second day of life, necessitating intubation. Clinical findings included facial dysmorphia, camptodactyly, skeletal anomalies, and generalized muscular hypotonia. High-frequency oscillation ventilation and surfactant administration yielded marginal improvement. On the third day of life, a severe pulmonary hypertensive crisis necessitated inhaled and systemic pulmonary vasodilators along with volume and catecholamine therapy. Whole exome sequencing revealed a homozygous mutation in the leukemia inhibitory factor receptor (*LIFR*) gene, consistent with Stüve–Wiedemann syndrome.

**Discussion/conclusion:**

The case underscores the importance of considering and prompting evaluation of rare genetic causes in the differential diagnosis of PPHN, especially when other abnormalities are present and conventional therapies prove inadequate. Therapeutic strategies must account for the different pathophysiology of primary PPHN including vascular remodeling, as seen in SWS, which may not respond to pulmonary vasodilators typically employed in secondary PPHN due to vasoconstriction. In this case, the patient responded well to treatment for primary PPHN, but the use of high-frequency oscillation ventilation and surfactant was not helpful.

## 1 Introduction

Persistent pulmonary hypertension of the newborn (PPHN) is a life-threatening condition and typically secondary to pathologies such as meconium aspiration syndrome or sepsis (1). It is characterized by abnormal pulmonary vasoconstriction and/or structural remodeling of the pulmonary vasculature. Primary or idiopathic PPHN refers to the absence of parenchymal lung disease and implies intrauterine pulmonary vascular remodeling (2). Although rare, primary PPHN should be considered in severe or therapy-resistant cases (2).

Stüve–Wiedemann syndrome (SWS) (#OMIM 601559) is a rare autosomal recessive disorder characterized by congenital skeletal dysplasia, hyperthermia, and swallowing difficulties (3). Cardiorespiratory manifestations include respiratory distress and pulmonary arterial hypertension, both described as poor prognosis factors in SWS (4, 5). SWS is more common in the United Arab Emirates and in consanguineous families, with a prevalence of 0.5 per 10,000 births (6).

In this report, we present a neonatal case of SWS with severe respiratory failure and PPHN, shedding light on the intricate diagnostic process, therapeutic interventions, and long-term outcomes.

## 2 Case description

**Figure 1:**
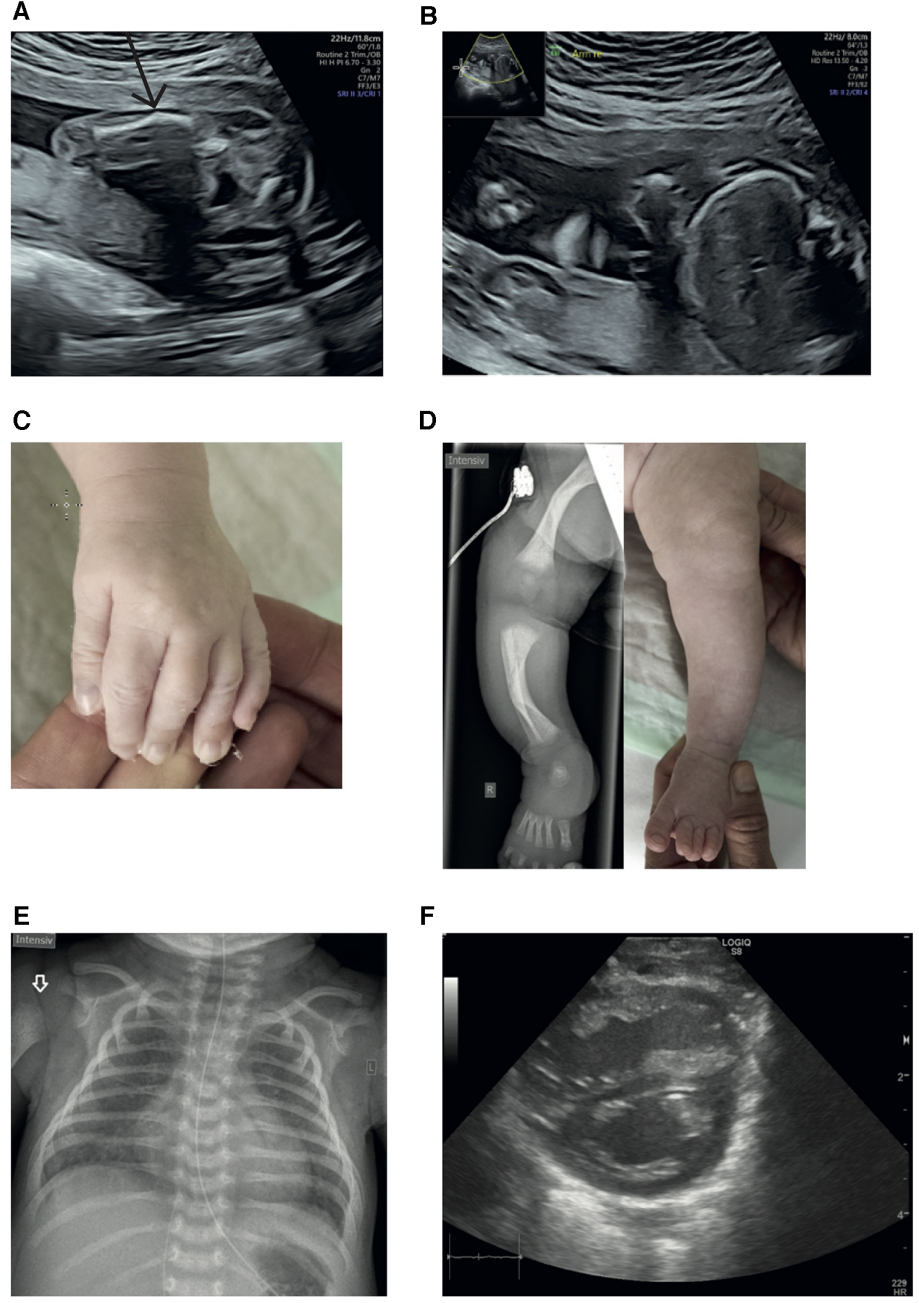
(**A**) Ultrasound at 22 + 1 weeks of gestation: left femur with a suspected fracture. (**B**) Ultrasound at 22 + 1 weeks of gestation: shortened right upper extremity. (**C**) Camptodactyly of digits II and V; extension was not possible. (**D**) Radiograph of the right lower limb and image of the left lower extremity illustrating bowing of the lower limb bones, affecting the tibia more than the femur (7–9). (**E**) Chest x-ray of the female full-term neonate displays a slightly enlarged heart and homogeneous ground-glass opacities of both lungs (in full-term neonates, the lungs are normally much more transparent). Note the missing ossification of the proximal humeral epiphysis in concert with an indistinct border of the metaphysis (white arrow). (**F**) Dilated right ventricle with D sign.

The patient, a full-term neonate, was admitted to our Neonatal Intensive Care Unit postnatally for respiratory distress syndrome. Notably, second-trimester prenatal ultrasound had earlier revealed multiple anomalies, including shortened and bowed limbs (Figures 1A,B), finger malposition, and intrauterine growth restriction. The 20-year-old primigravida had chosen not to pursue further prenatal diagnostics. Parental consanguinity was confirmed in the family history as the parents were cousins once removed, though without any indication of skeletal deformations.

The baby girl was delivered via cesarean section due to breech presentation, with Apgar scores of 5/8/8. Immediate respiratory distress required non-invasive ventilation.

On clinical examination, the patient exhibited various abnormal morphological features, including low-set ears, camptodactyly of digits II and V (Figure 1C), shortened and bowed extremities (Figure 1D), and bilateral flexion contractures of the arms and digit III.

On admission, the chest x-ray was consistent with mild respiratory distress syndrome (Figure 1E). Within the first day of life, the respiratory condition of the newborn deteriorated, prompting intubation, surfactant administration, and initiation of milrinone due to pulmonary hypertension confirmed by echocardiography (Figure 1F). Despite brief improvement, the condition of the patient rapidly worsened by the third day of life, resulting in a severe pulmonary hypertensive crisis (maximum oxygenation index of 33). Hemodynamic stabilization was achieved under treatment with inhaled nitric oxide (iNO), inhalative and intravenous iloprost, and extensive volume, catecholamine, and hydrocortisone therapy. A trial of high-frequency oscillation ventilation (HFOV) had an adverse effect on oxygenation. To maintain patency of the ductus arteriosus and thereby reduce right ventricular afterload when right-to-left shunt occurred, alprostadil was administered. Subsequently, the condition of the patient gradually improved, leading to successful extubation on the 14th day of life.

During the course of the hospital stay, the patient developed repeated self-limited episodes of hyperthermia, without signs of sepsis per laboratory results.

Neurological examination revealed generalized hypotonia alongside swallowing difficulties. Radiological examination of the lower limbs indicated bowing of the long bones with metaphyseal broadening, metaphyseal osteopenia, and diaphyseal cortical thickening (Figure 1D). The upper limbs were not radiographed because they did not show bowing or shortening.

In the face of the severe clinical course with refractory hypoxia, several differential diagnoses were evaluated simultaneously, including early initiation of whole exome sequencing (WES).

In the absence of evident secondary PPHN causes such as meconium aspiration or pulmonary malformations on chest x-ray, a connatal lung disease, e.g., alveolar capillary dysplasia, was one of the suspected diagnoses. However, this hypothesis was rejected when oxygenation improved under therapy over the initial days. Another differential diagnosis was hypophosphatasia, which is associated with shortening and bowing of limbs and respiratory complications. However, our patient did not meet the diagnostic criteria consisting of a deficiency of serum and bone alkaline phosphatase activity.

A severe vitamin D deficiency during pregnancy was considered as a cause of bone deformities given that both the mother and the infant had low vitamin D levels. However, this only explained some of the symptoms, and vitamin D deficiency normalized under substitution.

Finally, on the 19th day of life, WES revealed a homozygous pathogenic variant of the leukemia inhibitory factor receptor (*LIFR*) gene, which was consistent with a diagnosis of SWS (Table 1).

**Table 1:** *LIFR*: homozygous frameshift variant in *LIFR*; biallelic variants are associated with autosomal recessively inherited Stüve–Wiedemann syndrome/Schwartz–Jampel type 2 syndrome (OMIM #601559, ORPHA: 3206).

Gene	Variant	ClinVar	Incidence in gnomAD
*LIFR*	Hg19:chr5_38510800_T/TA NM_001127671.2:c756dup NP_001121143.1:p.Lys253Ter homozygous	Pathogenic (5)	0 heterozygous 0 homozygous

Long-term treatment with sildenafil orally alleviated pulmonary hypertension, with no evidence of the condition at 11 months of age. However, the patient faced challenges, exhibiting dystrophy and reliance on nasogastric tube feeding.

## 3 Discussion/conclusion

Skeletal dysplasias are rare and encompass a broad spectrum of more than 450 entities grouped into 42 categories based on radiographic, molecular, and biochemical criteria established by the International Skeletal Dysplasia Society (10). Prenatal ultrasound is a valuable tool for early diagnosis of skeletal disorders; however, the identification of rare syndromes remains challenging due to overlapping features and phenotypic heterogeneity (11). Genetic testing using next-generation sequencing can aid in identifying rare syndromes such as SWS antenatally, facilitating comprehensive peri- and postnatal management that takes into account potential complications, including pulmonary hypertension.

Fortunately, within the setting of a Level 3 perinatal center, swift action could be taken to identify and address PPHN in our patient, even though SWS was not diagnosed antenatally.

Initially described by Stüve and Wiedemann in 1971 (12), SWS was not recognized as a distinct condition until 2000, displaying significant similarities with a severe form of Schwartz–Jampel syndrome (7). It was only in 2004 that Dagoneau et al. shed light on the genetic and molecular roots, linking it to several mutations in the *LIFR* gene (#OMIM 601559) mapped on the 5p13 chromosome (13). Although it was initially regarded as lethal in infancy, subsequent reports have highlighted cases and series of childhood survivors with SWS (14). While skeletal dysplasia and autonomic disturbances are typical features, pulmonary hypertension, although less frequent, is associated with poor prognostic outcomes and a significant mortality rate of 63% in SWS (5). Notably, there is only one case report so far focusing in more detail on possible cardiovascular abnormalities in SWS. This report discusses two siblings with SWS who succumbed in the neonatal period due to severe pulmonary hypertension (4). While this report hints at the potential link between respiratory insufficiency and severe pulmonary hypertension due to pulmonary arterial wall medial hypertrophy causing early neonatal death in SWS, it falls short of delving into effective treatments for pulmonary arterial hypertension within this context.

**Figure 2:**
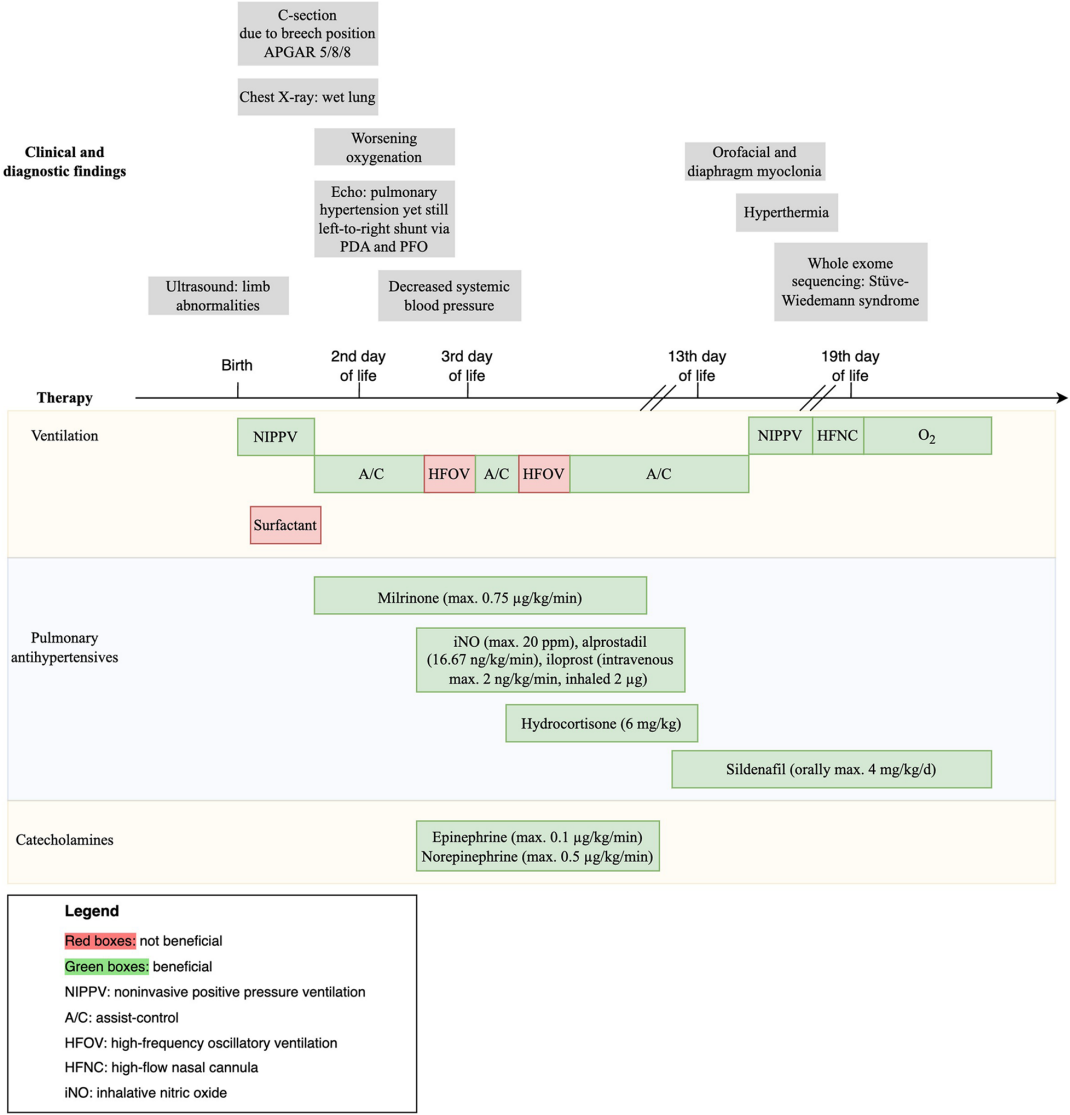
Case report timeline.

Against this backdrop, our case study steps in with a unique perspective, even though SWS was not diagnosed during the acute phase of pulmonary hypertension. The comprehensive timeline depicted in Figure 2 specifically outlines the tailored diagnostic and therapeutic strategies, along with the clinical findings, employed throughout the course of treatment.

Supportive management of PPHN entails maintaining normothermia, ensuring nutritional support, and providing sedation and analgesia as required (2). Optimal mechanical ventilation is essential to avoid under- or overinflation, which could exacerbate pulmonary vascular resistance. HFOV may benefit secondary PPHN but not idiopathic PPHN (15), consistent with our case, in which HFOV worsened oxygenation. Furthermore, as seen in our patient, surfactant administration, which is recommended when PPHN is secondary to surfactant deficiency or parenchymal lung disease (16), has little effect on primary PPHN patients.

In the realm of therapeutic interventions, inhaled pulmonary vasodilators, especially iNO, are typically considered first-line treatment options to reduce pulmonary vascular resistance in neonates (2). However, the pathophysiology differs between secondary and primary PPHN. In SWS and accordingly in primary PPHN, vascular remodeling appears to be the main driver of pulmonary hypertension (4), whereas in secondary PPHN, increased pulmonary vascular resistance predominantly originates from vasoconstriction. Consequently, iNO, which is a dilator of vascular smooth muscle, is more effective in secondary PPHN. Despite its temporary positive effects on oxygenation, the impact of iNO in our case was limited. Further treatment options include milrinone (17), sildenafil (18), prostaglandin E1 (19), and prostacyclin (20, 21). We started sildenafil while weaning iNO to avoid rebound. The effects of inhaled prostacyclin were limited and interfered with iNO, leading to its discontinuation. Hydrocortisone, often used as a rescue strategy (22), has produced favorable effects on oxygenation in cases involving meconium aspiration syndrome (23). In the case described here, hydrocortisone was administered without adverse reactions.

In general, the complexity of using multiple medications during the critical phase can complicate the determination of the most beneficial one. Further complicating matters, other features of SWS, including dysautonomia with hyperthermia, can pose challenges to management and often prompt unnecessary repeated laboratory evaluations to rule out infection, as seen in our patient. Again, early genetic testing to establish the diagnosis of SWS helps contextualize symptoms and optimize management.

In summary, skeletal abnormalities detected through prenatal ultrasound often lack specificity and should prompt early genetic evaluation using exome sequencing to establish the diagnosis, especially of rare conditions. Addressing skeletal anomalies promptly and pursuing additional testing leads to timely diagnoses, facilitating effective anticipatory treatment planning. The confluence of pulmonary hypertension, respiratory complications, and limb abnormalities could signify the presence of SWS. For patients with SWS, vigilance for the development of PPHN during postnatal adaptation is crucial, necessitating a clinical approach akin to primary PPHN management. Furthermore, episodes of hyperthermia, a hallmark of SWS, require special attention and consideration.

## Data Availability

The original contributions presented in the study are included in the article/Supplementary Material, further inquiries can be directed to the corresponding authors.
